# In Vitro Digestibility and Structural Evaluation of Pea Protein-Based Emulsion-Filled Gels Designed for Dysphagia-Friendly Nutrition

**DOI:** 10.3390/gels12040342

**Published:** 2026-04-19

**Authors:** Ieva Bartkuvienė, Viktorija Eisinaitė, Evren Golge, Vilma Petrikaitė, Daiva Leskauskaitė

**Affiliations:** 1 Department of Food Science and Technology, Kaunas University of Technology, LT-50254 Kaunas, Lithuania; viktorija.eisinaite@ktu.lt (V.E.); daiva.leskauskaite@ktu.lt (D.L.); 2 Department of Nanotechnology Engineering, Sivas Cumhuriyet University, Sivas 58140, Turkey; egolge@cumhuriyet.edu.tr; 3 Laboratory of Drug Targets Histopathology, Institute of Cardiology, Lithuanian University of Health Science, Sukilėlių pr. 13, LT-50162 Kaunas, Lithuania; vilmapetrikaite@gmail.com

**Keywords:** proteins, interfacial composition, emulsion-filled gel, dysphagia, in vitro digestion, microstructure, rheology

## Abstract

This study examined the structural, rheological, and digestive properties of plant-based emulsion-filled gels (EFGs) formulated for dysphagia-friendly nutrition. EFGs were created using a pea protein–κ-carrageenan (PP–κ-CAR) matrix that incorporated oil droplets stabilized by pea protein (EFG-PP), soy lecithin (EFG-PP/LEC), or mono-/diglycerides (EFG-PP/MDG). All formulations met the International Dysphagia Diet Standardisation Initiative Level 6 requirements and showed improved viscoelastic properties compared to the hydrogel control. The interfacial composition determined how the oil droplets influenced the gel network, with droplets in EFG-PP and EFG-PP/MDG contributing to greater reinforcement, whereas those in EFG-PP/LEC resulted in a weaker and more deformable structure. Among the formulations, EFG-PP/LEC demonstrated the most suitable properties for dysphagia management, including the lowest yield stress, softest texture, and highest protein hydrolysis (54%) and free fatty acid release (7.35 µmol/mL). These effects were associated with weaker oil–matrix interactions and greater enzymatic accessibility. The findings highlight the importance of interfacial design in tailoring EFG structure and digestibility for safe, energy-dense diets for individuals with dysphagia.

## 1. Introduction

Swallowing is a vital physiological process that involves the coordinated and sequential movement of food or liquids from the mouth through the pharynx and into the esophagus [1,2]. Dysphagia, characterized by impairments at one or more stages of this process, can affect individuals of all age groups [3,4]. This condition can greatly reduce the quality of life and increase the risk of aspiration or choking incidents. Repeated choking episodes may lead to a fear of swallowing, resulting in decreased food intake and subsequent malnutrition and frailty [5]. Managing dysphagia involves creating diets tailored to the severity of the impairment, ensuring safety, ease of swallowing, and adequate nutrition with high energy density and sufficient protein levels to meet individual dietary needs [6].

In recent years, plant-based emulsion-filled gels (EFGs) have become promising texture-modified foods for people with dysphagia. EFGs are usually made by dispersing emulsified oil droplets within the gel matrix [7]. Including oil increases both calorie density and lubrication, which helps manage malnutrition and improves oral processing [8,9]. Proteins from sources such as peas, soybeans, wheat, and potatoes [10] are used in plant-based EFG formulations. Among these, pea proteins have gained particular attention due to their balanced amino acid profile, low allergenicity, non-GMO status, and widespread consumer acceptance, making them especially suitable for food applications [11,12]. However, because of their limited functional properties, especially gelling and emulsifying abilities, plant proteins are often combined with polysaccharides such as konjac glucomannan [13], inulin [14], κ-carrageenan [15], agar [16], or pectin [17] to achieve the desired rheological and textural properties [18,19].

The nature of the interfacial layer surrounding oil droplets determines whether droplets behave as active or inactive fillers within EFGs. Active fillers are oil droplets whose interfacial layers interact with the surrounding gel matrix through physical entanglements, electrostatic attractions, or specific molecular interactions, allowing stress transfer between the droplets and the matrix and reinforcing the gel network. In contrast, inactive fillers exhibit limited or no interfacial interactions with the gel matrix. Such droplets primarily act as inert inclusions that disrupt matrix continuity and may weaken gel structure [18,20]. In emulsions, pea proteins can act as the primary interfacial stabilizers due to their amphiphilic nature. Their hydrophobic and hydrophilic groups spontaneously adsorb at the oil–water interface, forming a dense and flexible interfacial film [21]. In addition to proteins, low-molecular-weight emulsifiers are frequently incorporated into emulsion-based systems to further tailor interfacial properties and emulsion characteristics. Food-grade emulsifiers such as soy lecithin and mono- and diglycerides of fatty acids (MDG), which are widely used low-molecular-weight surfactants in food systems, are commonly employed to tailor interfacial characteristics in emulsions [22,23]. Moreover, the protein molecules within this interfacial film can synergistically interact with the other emulsifiers, thereby modulating the emulsion’s rheological behavior and textural properties [24].

To date, several plant protein EFGs have been developed; however, their evaluations have mostly focused on delivery performance [25,26,27] or structural characterization and stability [9,28,29], with limited consideration for their nutritional role as a source of protein and energy. Studies on plant-based EFG protein digestibility showed relatively low levels of protein hydrolysis at the end of intestinal digestion. For example, X. Li et al. [15] demonstrated that in pea protein–κ-CAR EFGs, the degree of protein hydrolysis after 4 h of intestinal digestion did not exceed ~10% and decreased further with increasing polysaccharide concentration. Similar trends were reported for other plant protein–polysaccharide EFG systems, where modifications in interfacial composition altered filler activity and digestion kinetics, but still resulted in limited proteolysis at the end of digestion [30].

Therefore, in this study, we aimed to develop calorie-dense, plant-based emulsion-filled gels (EFGs) with different interfacial compositions and to evaluate their effect on structural and digestive properties, as well as their suitability for a dysphagia diet. We expanded our previously developed heat-induced pea protein–κ-CAR (PP-κ-CAR) hydrogel systems [31] by incorporating oil stabilized by proteins present in the PP-κ-CAR hydrogel, soy lecithin, and mono- or diglycerides of fatty acids (MDG). Three EFG formulations were developed, including EFG-PP, EFG-PP/LEC, and EFG-PP/MDG, each containing 20% oil, 10% PP, and 0.48% κ-CAR. Their properties were compared to those of the PP-κ-CAR hydrogel containing equivalent PP and κ-CAR concentrations. The suitability of these formulations for a dysphagia diet was evaluated through rheological and textural analysis, compliance with IDDSI tests, and in vitro digestibility.

## 2. Results and Discussion

### 2.1. Characterization of Emulsion-Filled Gels

#### 2.1.1. Microstructural Properties

**Figure 1:**
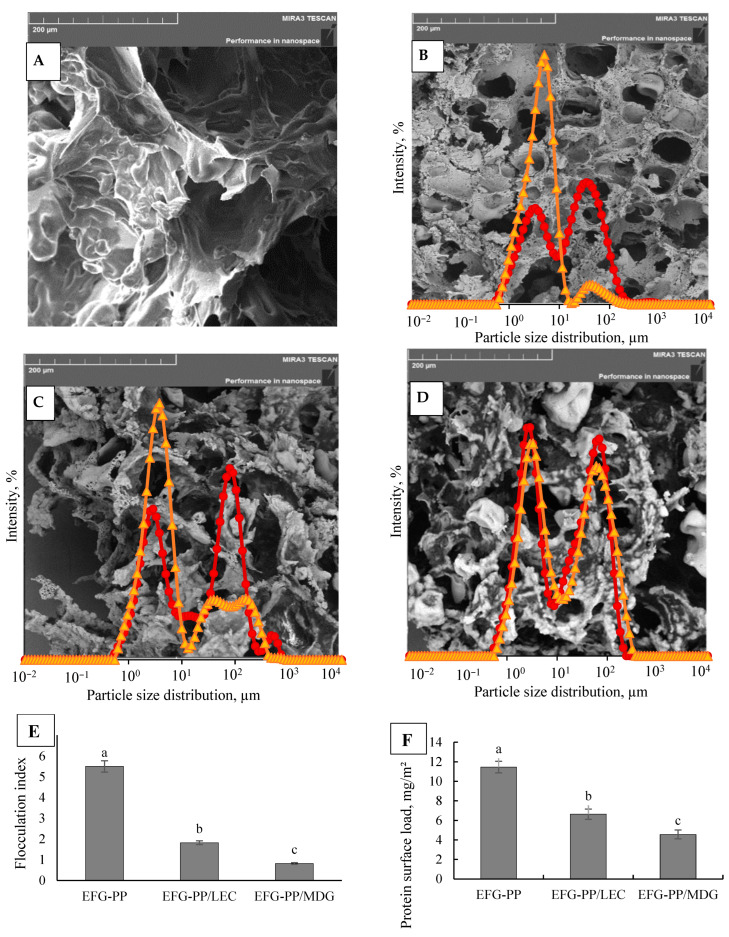
SEM micrograph of PP-κ-CAR hydrogel (**A**); SEM and particle size distribution of EFG-PP (**B**), EFG-PP/LEC (**C**), and EFG-PP/MDG (**D**); flocculation index (**E**); and protein content adsorbed at the oil–water interface (**F**). Triangular markers in (**B**–**D**) indicate particle size distribution of EFGs in the presence of SDS, and circular markers indicate particle size distribution of EFGs diluted in water. Different letters indicate significant differences (*p* < 0.05).

To assess the impact of differently stabilized oil droplets on the gel microstructure, SEM images of the EFGs were compared to those of a similar composition system that did not contain oil (HG). Micrographs were captured at 500× magnification, and representative microstructures are shown in Figure 1A–D. The HG sample (Figure 1A) exhibited a dense and continuous gel network, while the EFGs (Figure 1B–D) displayed a more porous microstructure. The pores observed in the EFGs could be associated with regions previously occupied by oil droplets that were removed during sample preparation for SEM analysis. In the EFG-PP sample (Figure 1B), where oil droplets were stabilized solely with PP, the pores were of well-defined circular shape. The incorporation of lecithin or mono-/diglycerides (MDG) into the EFG formulations influenced the pore size and morphology of the gel matrix, with EFG-PP/LEC and EFG-PP/MDG showing larger, more irregularly shaped pores (Figure 1C,D).

To further evaluate the influence of different emulsifiers on oil droplet characteristics, the particle size distribution of emulsion-filled gels was analysed after dilution in water (Figure 1B,C, circular red markers) and in the SDS, which disrupts the bridges between flocculated oil droplets (Figure 1B,C, triangular orange markers). In the samples without SDS, a broad multimodal oil droplet size distribution was observed for all three systems, indicating the presence of both small and large oil particles. Two major peaks were observed for all EFGs: at 3 and 35 µm for EFG-PP, 4 and 80 µm for EFG-PP/LEC, and 3 and 70 µm for EF-PP/MDG, with the second peak exhibiting higher intensity. In the presence of SDS, the droplet size distribution of EFG-PP and EFG-PP/LEC shifted towards smaller particle sizes, with the main peak at 3–4 µm, suggesting that the initial larger peaks in these EFGs result from flocculation rather than the presence of large oil droplets [32,33]. In contrast, EFG-PP/MDG exhibited only minor SDS-induced shifts, indicating limited flocculation. In this sample, greater polydispersity likely arose from the presence of diglycerides, which favor larger droplet formation [34].

Consistent with these observations, the calculated flocculation index (Figure 1E) was the highest in EFG-PP (FI = 5.5), followed by EFG-PP/LEC (FI = 1.82) and EFG-PP/MDG (FI = 0.82). The FI results are consistent with interfacial protein surface load (Figure 1F), where the highest protein surface content was determined in EFG-PP (11.5 mg/m^2^), while EFG-PP/LEC and EFG-PP/MDG systems revealed a significantly lower protein load of 6.6 mg/m^2^ and 4.56 mg/m^2^, respectively. As gels were heated during gel preparation, pea proteins adsorbed at the oil–water interface aggregate [35] and formed interdroplet protein bridges between droplets [32]; therefore, higher protein content yields higher FI.

#### 2.1.2. Rheological Properties

- Temperature sweep

**Figure 2:**
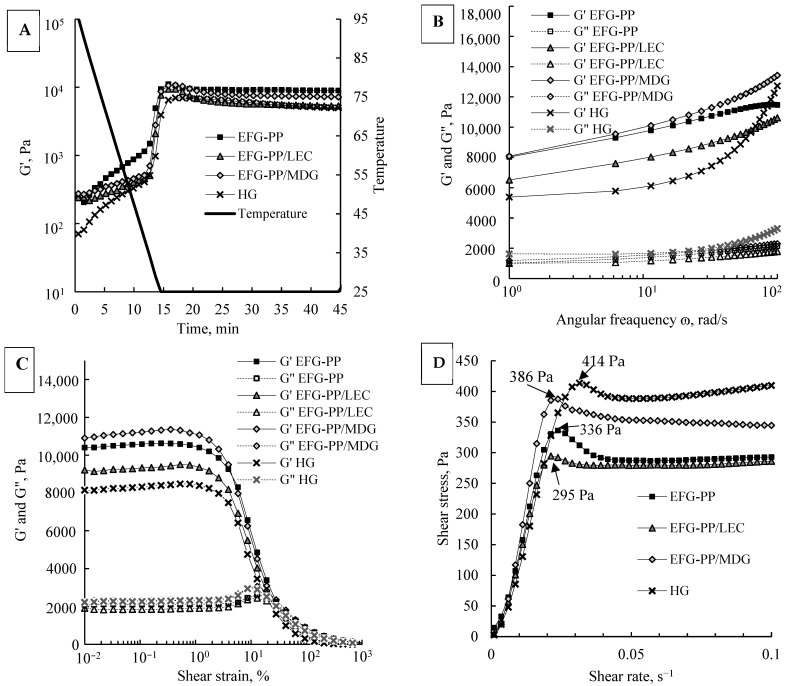
Temperature sweep (**A**), frequency sweep (**B**), strain-dependent viscoelasticity (**C**), and yield point (**D**) of HG and different EFGs.

To evaluate differently stabilized oil droplets’ effect on the EFG gelation behavior, the storage modulus (G′) values of gels during the dynamic temperature ramp from 95 °C to 25 °C were measured and are presented in Figure 2A. The gelation curve consists of three parts: a gradual increase in G′ values (0–12 min), followed by a sharp increase (12–15 min), and a plateau phase (15–45 min). As the temperature decreased from 95 °C to ~38 °C (0–12 min), EFG-PP and HG systems revealed a steeper increase in G′ values than EFG-PP/LEC and EFG-PP/MDG. This difference could be related to oil droplets acting as active or inactive fillers in the system, as oil droplets, which function as active fillers, can accelerate the crosslinking rate and gelation process through the interactions with the gel network [36]. These observations are consistent with SEM results, where EFG-PP revealed smaller and more uniform pores than the other systems, which is associated with faster gelation. As the temperature decreased further to 25 °C (12–15 min), all systems exhibited a sharp increase in G′ values, indicating the formation of gel.

At the end of the plateau phase, EFG-PP and EFG-PP/MDG showed significantly higher G′ values (8925 ± 357 Pa and 7192 ± 213 Pa, respectively) than EFG-PP/LEC (5308 ± 105 Pa) and HG (4984 ± 102 Pa), indicating that the interfacial composition of oil droplets influenced the final gel strength. In all EFG systems, proteins were present at the droplet interface (Figure 1F), suggesting that oil droplets could potentially interact with the surrounding pea protein–κ-carrageenan network. In the EFG-PP system, the higher gel strength compared with HG suggests that protein-stabilized oil droplets contributed to reinforcement of the gel network through interactions between proteins at the droplet interface and proteins in the pea protein–κ-carrageenan network [37]. A similar strengthening effect was observed in the EFG-PP/MDG system, which showed a markedly higher final G′ value than HG despite a slower increase in G′ during the early stage of cooling. In contrast, EFG-PP/LEC showed a markedly lower final G′ value than EFG-PP and EFG-PP/MDG, although the amount of protein adsorbed at the interface was higher in EFG-PP/LEC than in EFG-PP/MDG. This indicates that gel strengthening depended not only on the amount of protein adsorbed at the droplet interface, which could enable interactions with the surrounding gel network, but also on the functionality of the low-molecular-weight emulsifier. MDG has been reported to act not only as an emulsifier but also as an oleogelator [38], promoting the solidification of oil droplets within the gel network, which may further reinforce gel structure [39,40]. Overall, these results indicate that oil droplets in EFG-PP likely behaved as active fillers, contributing to gel reinforcement through protein-mediated interfacial interactions with the surrounding pea protein–κ-carrageenan network. In EFG-PP/MDG, the elevated final G′ suggests a strong reinforcing contribution of the dispersed phase; however, this effect cannot be attributed exclusively to active-filler behavior, because MDG may also strengthen the system by structuring or partially solidifying the oil droplets. In contrast, oil droplets in EFG-PP/LEC showed a weaker contribution to gel strengthening, consistent with more inactive-filler-like behavior.

- Frequency sweep

To assess the gels’ mechanical stability under oscillatory conditions, the frequency sweeps of different gel systems, showing the storage modulus (G′) and loss modulus (G″) as a function of angular frequency, were measured (Figure 2B). Results showed that all systems displayed gel-like behavior (G′ > G″) across the entire frequency range. At 1 rad/s, HG had the lowest G′ (5382 ± 86 Pa) and the highest G″ (1627 ± 26 Pa), consistent with a weaker and more viscous network. In contrast, EFG-PP, EFG-PP/LEC, and EFG-PP/MDG exhibited significantly higher G′ values (6515 ± 104 Pa, 8037 ± 224 Pa, and 8091 ± 212 Pa, respectively), reflecting stronger and more elastic structures. Higher elasticity, particularly in EFG-PP and EFG-PP/MDG, is consistent with active filler effects and may help support bolus cohesion during oral processing [41]. The observed increase in G′ and G″ values with increasing frequency indicates that the structures became stiffer at higher frequency. This effect occurs when the molecules in the gel network have less time to rearrange themselves during each cycle of applied stress at higher frequencies, resulting in stronger resistance to deformation [42]. The frequency dependence was markedly lower in EFGs, suggesting that incorporation of oil droplets, regardless of the emulsifier used, improved the gel’s stability when subjected to stress.

- Strain-dependent viscoelasticity

To determine the linear viscoelastic region (LVR) of gels, G′ and G″ were measured as a function of increasing strain (Figure 2C). The critical strain of LVR was identified as 2.61% for all samples, based on the method described by Lin et al. [43], which defines it as the point where G′ deviates by more than 5% from its prior value. Within the LVR, the gels exhibited solid-like behavior, characterized by constant values of G′ and G″. Once the applied strain exceeded the critical strain, a sharp decline in G′ was observed, indicating the onset of structural breakdown and a transition from the solid to the liquid-like state. This indicates that all gels remained cohesive under low strain, but transitioned to a flowable state when sufficient force was applied.

- Shear yield stress

Yield stress, the minimum stress required for the material to start flowing, provides information on how food bolus will behave during oral processing, as food cannot be effectively propelled by the tongue if the applied stress stays below the yield point [4,44]. In this study, shear yield stress of gels was determined as the peak in shear stress formed during an increasing strain rate (Figure 2D). HG reached peak shear stress at 0.03 s^−1^ shear rate and exhibited the highest yield stress value (414 ± 9 Pa). On the contrary, EFGs peaked at 0.02 s^−1^, and showed lower yield stress values of 386 ± 6 Pa, 336 ± 11 Pa, and 296 ± 10 Pa for EFG-PP/MDG, EFG-PP, and EFG-PP/LEC, respectively. Foods with lower yield stress are associated with easier manipulation during oral processing, requiring less chewing effort and improving safety and comfort for individuals with swallowing impairments [6,9,45]. Thus, all EFG systems showed lower yield stress than HG, suggesting potential advantages for dysphagia-friendly formulations. Among them, EFG-PP/LEC exhibited the lowest yield stress.

### 2.2. Gel’s Suitability for Dysphagia Diet

The results of IDDSI testing confirmed that all tested samples met the criteria for Level 6 (Soft & Bite-sized), as they did not recover their shape after fork pressure was released (Table S1). No visual differences were observed among the samples. Although IDDSI testing is useful in clinical practice to confirm that products are suitable for patients with dysphagia, the fact that all samples were classified into the same level shows that this method may not be sensitive enough to detect differences in mechanical texture properties between formulations. This means that IDDSI alone cannot distinguish between formulations that may differ in important mechanical properties, such as hardness, cohesiveness, adhesiveness or chewiness, which can influence patient comfort, oral processing, and overall acceptability. Thus, combining IDDSI with instrumental texture profile analysis (TPA) offers a more detailed and reliable way to describe and compare texture-modified foods [46,47].

In this study, TPA was used to measure the hardness, chewiness, adhesiveness, springiness, and cohesiveness of the gels, and the results are summarized in Table 1. Hardness refers to the force required to compress food between the tongue and palate [29]. Among the samples, EFG-PP exhibited the highest hardness (509 g), exceeding that of EFG-PP/MDG (494 g) and hydrogel control (489 g). In contrast, the EFG-PP/LEC system showed a significantly softer texture (429 g), which was due to weaker interactions between lecithin-coated oil droplets and the protein–polysaccharide matrix. These trends are consistent with the Gʼ values obtained from rheological measurements (Figure 2A) and align with prior reports on oil droplet–matrix interactions enhancing gel strength [48]. The hardness values obtained are comparable to other mild dysphagia-appropriate gels, including egg yolk and CMC-based emulsion gels [49] and shiitake mushroom-based gels [44]. In addition, chewiness, which represents the energy needed to masticate food into a soft and swallowable bolus [29], increased significantly in EFG samples (85.79–105.28 g) compared with the HG control (48.99 g). As dysphagia-friendly foods should require minimal mastication effort, lower chewiness values are considered more favorable for swallowing safety and comfort [6]. From this perspective, EFG-PP/LEC showed the most favorable chewiness among the tested EFG systems.

Cohesiveness, reflecting the ability of gel to maintain structural integrity during compression, also increased with the addition of oil, with values increasing from 0.15 in HG to 0.25–0.30 in EFGs. Higher cohesiveness suggests that EFGs are better able to maintain their internal structure during deformation, preventing them from disintegration into smaller fragments during oral processing [37,50]. Moreover, springiness, describing the elastic recovery of the gels after compression, ranged from 0.67 to 0.80 and was significantly higher in the EFG-PP/LEC system than in the other samples. This implies enhanced elastic recovery in EFG-PP/LEC, which can support bolus formation during mastication [37,51].

Finally, adhesiveness, defined as the work required to overcome the attractive forces between the sample and the instrument probe simulating human teeth [50], reflects the sample’s stickiness. Oil addition increased the adhesiveness, as the HG sample showed significantly lower adhesion values (−41.78 g·s) compared to EFG systems (−72.77 to −91.43 g·s). These values exceed those reported for other dysphagia-friendly diets [6,52], suggesting that the tested gels may present greater stickiness. This is relevant because foods with high adhesiveness may adhere to the teeth and tongue, posing a risk of choking [53,54].

Overall, oil incorporation modulated the textural properties of the gels, enhancing hardness, cohesiveness, and springiness, which may support bolus integrity during oral processing. However, the increases in chewiness and adhesiveness highlight potential limitations related to mastication effort and stickiness in dysphagia-oriented foods. Among the emulsion-filled gels, EFG-PP/LEC showed lower hardness and chewiness together with higher springiness and cohesiveness, which may be advantageous for Level 6 dysphagia applications. At the same time, its higher adhesiveness should also be considered, as increased stickiness may present an additional challenge during oral processing and swallowing. Nevertheless, texture and rheological measurements alone cannot fully predict how these gels would behave during oral processing, and further in vitro or in vivo swallowing studies are required to better assess their safety and suitability for dysphagia management.

### 2.3. Degradation of EFGs During In Vitro Digestion

In this study, in vitro digestion was performed using the standardized INFOGEST protocol to examine how incorporating oil with different interfacial compositions into the PP-κ-CAR hydrogel system impacts gel digestibility. CLSM images of digesta, particle size distribution, degree of hydrolysis, and free fatty acid release were measured at different gastric and intestinal digestion stages to evaluate these effects.

#### 2.3.1. Particle Size Distribution and Microstructure of EFGs During In Vitro Digestion

**Figure 3:**
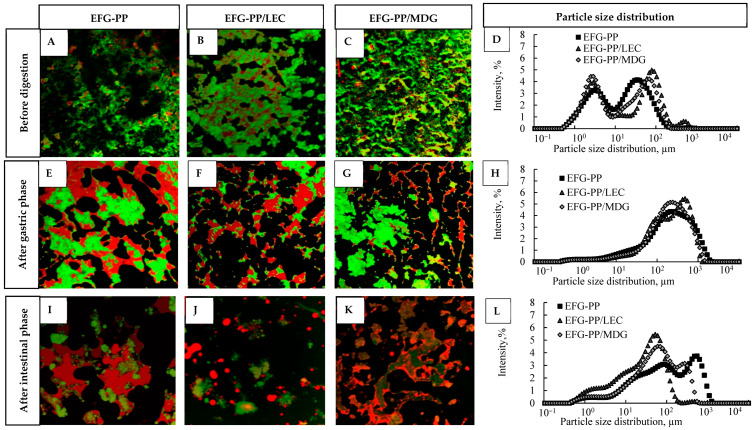
CLSM images of EFGs at different stages of in vitro digestion: before digestion (**A**–**C**), after 2 hours of gastric phase (**E**–**G**), and after 4 h of intestinal digestion (**I**–**K**). The corresponding particle size distributions are shown in (**D**,**H**,**L**) for the samples before digestion, after 2 h of the gastric phase, and after 4 h of the intestinal phase, respectively.

Structural changes during in vitro digestion were visualised using CLSM, with proteins stained green and lipids stained red, as shown in Figure 3. Before digestion, all EFG systems exhibited a porous network structure with a polydisperse distribution of oil droplets throughout the pea protein and κ-CAR network (Figure 3A–C). After two hours of gastric digestion, substantial fragmentation of the gel matrix was observed, together with aggregated protein particles of varying sizes and large flocculated and coalesced oil droplets (Figure 3E–G). Notably, in the EFG-PP system (Figure 3E), the oil droplets stabilized by PP formed larger flocs than those in EFG-PP/LEC (Figure 3F) and EFG-PP/MDG (Figure 3G), whereas the smallest protein network particles were observed in the EFG-PP/LEC system. The fragmented EFG particles were further broken down during the intestinal phase into smaller protein and oil particles (Figure 3I–K). However, even after 4 h of intestinal phase digestion, both protein fragments and oil droplets remained visible, indicating that complete proteolysis and lipolysis had not occurred. This limited digestibility is consistent with findings from other studies on EFGs, which have reported undigested protein gel particles and lipid droplets at the end of the simulated gastrointestinal digestion [15,30]. Additionally, it was observed that in the EFG-PP and EFG-PP/MDG systems, oil droplets remained associated with the protein gel particles. In contrast, in EFG-PP/LEC, most oil droplets were released from the gel matrix due to weaker interactions with pea protein–κ-CAR gel.

Similar to CLSM results, the particle size distribution revealed the same tendency. Before digestion, the samples displayed a polydisperse distribution of oil droplets, which by the end of the gastric phase shifted toward larger particle sizes, with a primary peak in the 240–480 µm range. Notably, unlike EFG-PP and EFG-PP/MDG, the EFG-PP/LEC system exhibited a bimodal distribution with overlapping peaks, including an additional peak around 100 µm, suggesting the presence of smaller oil droplets. By the end of the intestinal phase, a multimodal particle size distribution was observed, shifting toward smaller particles. This indicates droplet breakdown due to lipolysis. Among the systems, EFG-PP/LEC showed a greater shift towards smaller particle sizes, suggesting a faster rate of lipolysis.

#### 2.3.2. Degree of Protein Hydrolysis

**Figure 4:**
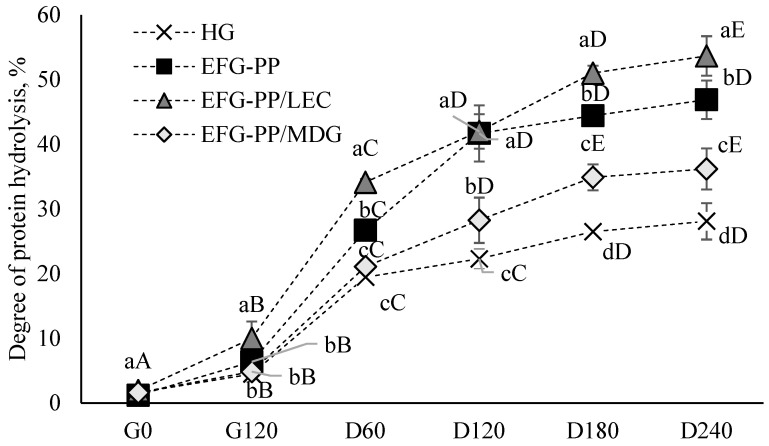
Degree of protein hydrolysis of different gel systems at specific time points during the gastric phase (0 and 120 min) and duodenal phase (60, 120, 180, and 240 min). Different letters indicate significant differences among tested samples (*p* < 0.05); lowercase—among different samples, uppercase—among different digestion points.

The in vitro gastrointestinal rate of protein hydrolysis for different EFGs and HG is shown in Figure 4. To evaluate how the incorporation of oil with different interfacial compositions affects protein digestibility, a pea protein–κ-CAR hydrogel (HG) with similar protein and κ-CAR concentrations was used as a control. During the gastric phase, only a small degree of protein hydrolysis (4–10%) was observed across all samples, followed by a marked increase during the intestinal phase. This trend reflects the enzymatic action of pepsin, which preferentially hydrolyses peptide bonds involving hydrophobic or aromatic amino acids, producing larger peptides in the stomach phase that are further degraded by pancreatin in the small intestine [55]. 

Oil incorporation into the HG system enhanced protein digestibility, with EFG systems showing a significantly higher degree of protein hydrolysis at the end of in vitro digestion (36–54%) than HG (28%). This improvement could be due to oil droplets disrupting the homogeneous pea protein–κ–CAR network, resulting in a looser and more porous structure, that was more accessible to digestive enzymes. However, the extent of protein hydrolysis also differed among the tested EFG systems, with the highest value observed for EFG-PP/LEC, suggesting that interfacial composition played an important role in digestion behavior.

In EFG-PP, interfacially adsorbed pea proteins acted as active fillers, reinforcing the gel network and promoting the formation of a denser microstructure with smaller pores, as observed in the SEM images (Figure 1B). Such a structure could hinder the diffusion of digestive enzymes into the gel matrix and reduce their access to protein substrates, thereby slowing protein hydrolysis and lowering in vitro protein digestibility [56]. In EFG-PP/MDG, the lower protein digestibility may additionally be related to the oil-structuring ability of MDG, which promotes the formation of partially crystallised or gelled oil droplets. Previous studies have shown that MG-induced structuring strengthens emulsion gels and that oleogelation delays digestive breakdown [57]. Therefore, in the present system, the structured oil droplets may have hindered matrix disintegration and enzyme penetration, indirectly reducing protein accessibility to digestive enzymes. By contrast, the LEC-stabilized system appeared to form a structure that was less resistant to disintegration. Its lower hardness may have contributed to this behavior, as EFG-PP/LEC exhibited the lowest hardness among the tested systems (429 g; Table 1), consistent with previous findings that softer gels disintegrate more readily during digestion [58]. This is in line with the assumption that the oil droplets in EFG-PP/LEC acted as inactive fillers, thereby weakening the gel network and promoting gel disintegration, as reflected by the smaller gel particles observed after the gastric phase (Figure 3F). Such smaller particles are known to undergo faster digestion due to more efficient pepsin diffusion [59].

Compared with values reported for other gelled protein systems [15,30], the final degree of hydrolysis observed in the EFGs was relatively high, indicating that these structured systems remained sufficiently accessible to digestive enzymes despite their gelled nature. Overall, these findings suggest that both oil incorporation and interfacial composition played important roles in modulating gel structure, disintegration behavior, and, consequently, protein digestibility.

#### 2.3.3. Release of Free Fatty Acids

**Figure 5:**
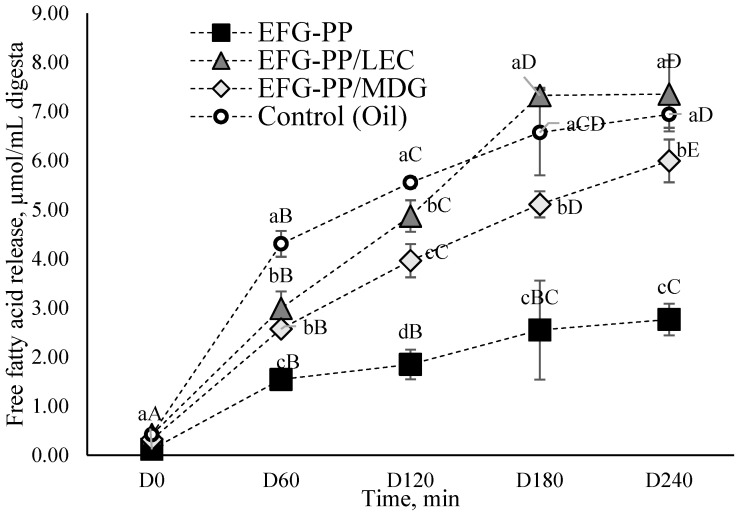
Release of free fatty acids from different gel systems at specific time points during the duodenal phase (0, 60, 120, 180, and 240 min). Different letters indicate significant differences among tested samples (*p* < 0.05); lowercase—among different samples, uppercase—among different digestion points.

Figure 5 shows the release of free fatty acids (FFA) over a 240 min intestinal digestion period for different EFG systems, compared to an unstructured oil sample used as the control. In the early stages of intestinal digestion, the control sample demonstrated the highest FFA release, indicating that free oil was more readily accessible to lipase enzymes. This observation aligns with the interfacial nature of lipid hydrolysis, which relies on the adsorption of pancreatic lipase to the surface of the oil droplets, where it catalyzes the breakdown of triacylglycerols into free fatty acids and mono- and diglycerides [30]. Bile salts aid this process by displacing surface-active compounds and lipid hydrolysis products from the oil droplets’ interface, forming mixed micelles that solubilize the lipolysis products and further allow access for lipase [60]. This explains why lipid digestion started at a slower rate in EFG systems, since additional time was required for bile salts to displace the interfacial layer surrounding oil droplets, thereby delaying lipase access and lipid hydrolysis.

It was evident that the interfacial composition was a critical factor determining the rate of lipid hydrolysis of EFG systems. The use of additional emulsifiers, lecithin and MDG, significantly increased the FFA release, with EFG-PP/LEC showing the highest amount by the end of the intestinal phase (7.35 µmol/mL), closely matching the value observed for the control sample. The EFG-PP/MDG revealed a slightly lower amount of FFAs (5.99 µmol/mL), while in the EFG-PP system, FFA release was the lowest, reaching only 2.76 µmol/mL by the end of digestion. The enhanced lipid hydrolysis in the EFG-PP/LEC and EFG-PP/MDG systems is attributed to the presence of non-ionic surfactants that inhibit the flocculation of protein-coated droplets during digestion, thus increasing the droplet dispersion and improving enzyme accessibility [24].

In the case of EFG-PP/LEC, as discussed in Section 2.3.2, weaker interactions between the oil droplets and the gel matrix, together with the softer gel structure, promoted faster disintegration and earlier release of oil droplets from the matrix, thereby facilitating access for bile salts and lipolytic enzymes [58,61]. In addition, Naso et al. [62] suggested that peptides released during gastrointestinal digestion of proteins may interact with bile salts and potentially increase the solubilization of lipolysis products. Since EFG-PP/LEC exhibited the highest degree of hydrolysis, this mechanism may also have contributed to the enhanced lipolysis observed in this system. The significantly lower release of FFA in the EFG-PP system may be the result of the formation of disulfide bonds and hydrophobic interactions at the oil–water interface, which occur when globular proteins unfold and expose their non-polar groups [24]. These interactions can strengthen the interfacial layer, making it more resistant to displacement by the bile salts.

## 3. Conclusions

This study examined how different interfacial compositions affect the structural, rheological, and digestive properties of emulsion-filled gels (EFGs) designed to manage dysphagia. Three EFGs were formulated using a pea protein–κ-carrageenan (PP-κ-CAR) hydrogel matrix with incorporated oil droplets, each stabilized by a different emulsifier: pea protein (EFG-PP), soy lecithin (EFG-PP/LEC), and mono- and diglycerides (EFG-PP/MDG). The nature of the emulsifier significantly modulated how the incorporated oil droplets contributed to the mechanical properties of the hydrogel network, with droplets in EFG-PP and EFG-PP/MDG promoting greater reinforcement, whereas droplets in EFG-PP/LEC showed a weaker reinforcing effect and a lower final G′ value. This, in turn, affected both the textural and in vitro digestibility characteristics of the resulting gels. All EFG formulations met the International Dysphagia Diet Standardisation Initiative (IDDSI) level 6 criteria, as determined by the fork test, and exhibited enhanced viscoelastic properties relative to a control PP-κ-CAR hydrogel lacking an oil phase. Among the tested systems, EFG-PP/LEC showed lower hardness and chewiness together with higher springiness and cohesiveness, which may be advantageous for Level 6 dysphagia applications. However, its higher adhesiveness should also be taken into account, as increased stickiness may pose an additional challenge during oral processing and swallowing. Therefore, although the EFG-PP/LEC formulation appeared to be the most promising, further sensory, in vitro, and in vivo swallowing studies are required to confirm its practical suitability for dysphagia management.

In vitro digestion assays revealed that EFG-PP/LEC displayed the highest degree of protein hydrolysis (54%) and free fatty acid release (7.35 µmol/mL), likely due to its softer texture and the weaker interaction between the oil droplets and the hydrogel matrix. These results highlight the important role of interfacial design in modulating the structure–digestibility relationship in plant-based EFGs, providing valuable insights for the development of tailored food products for individuals with dysphagia.

## 4. Materials and Methods

### 4.1. Materials and Chemicals

Pea protein isolate (80% protein content) was sourced from My Protein (Manchester, UK). κ-Carrageenan (κ-CAR, CAS No. 9000-07-1), potassium chloride (KCl, CAS No. 7447-40-7), potassium dihydrogen phosphate (KH_2_PO_4_, CAS No. 7778-77-0), sodium chloride (NaCl, CAS No. 7647-14-5), magnesium chloride hexahydrate (MgCl_2_·6H_2_O, CAS No. 7791-18-6), ammonium carbonate ((NH_4_)_2_CO_3_, CAS No. 506-87-6), and calcium chloride dihydrate (CaCl_2_·2H_2_O, CAS No. 10035-04-8) were purchased from Sigma-Aldrich (Steinheim, Germany). Bile extract porcine (B8631), lipase from porcine pancreas (type II, 100–500 U/mg, L3126), pepsin A (600–1200 U/mg, 77160), and pancreatin (P1625) were obtained from Sigma-Aldrich (Steinheim, Germany). Sodium dodecyl sulphate (SDS, CAS No. 151-21-3), fluorescamine (CAS No. 38183-12-9), and bovine serum albumin (BSA, CAS No. 9048-46-8) were purchased from Sigma-Aldrich (Steinheim, Germany). The free fatty acids assay kit (MAK466) was also obtained from Sigma-Aldrich. Canola oil was purchased from a local food retailer. Mono- and diglycerides of fatty acids (MDGs; food-grade emulsifier mixture) were procured from Puratos (Bijgaarde, Belgium). Fluorescein-5-isothiocyanate (FITC, CAS No. 3326-32-7) and Nile Red (CAS No. 7385-67-3) were obtained from Fluorochem (Derbyshire, UK), and Coomassie Brilliant Blue G-250 (CAS No. 6104-58-1) was purchased from Labochema (Vilnius, Lithuania). Soy lecithin (food-grade phospholipid mixture) was kindly provided by Alvas Group (Kaunas, Lithuania). All chemicals were of analytical grade unless otherwise stated.

### 4.2. Sample Preparation

To prepare the emulsion-filled pea protein–κ-CAR systems, pea protein (12.5% *w*/*w*), κ-CAR (0.6% *w*/*w*), and KCl (0.1% *w*/*w*) were dispersed in distilled water and allowed to hydrate for 2 h under continuous stirring using a magnetic stirrer at 1500 rpm (MSH-20D, Witeg, Wertheim, Germany). The hydrated mixture was subsequently maintained in a 95 °C water bath for 30 min (Wisd WiseBath, WITEG Labortechnik, Wertheim, Germany). After heating, the mixture was homogenized at 15,000 rpm for 2 min using ULTRA-TURRAX T 18 digital homogenizer (IKA, Staufen, Germany) together with one of three pre-heated oil phases: (1) oil without emulsifiers (EFG-PP), (2) oil with 1% soy lecithin (EFG-PP/LEC), or (3) oil containing 2% MDG (EFG-PP/MDG). The hydrogel-to-oil ratio was 4:1, resulting in gel formulations containing 10% protein and 20% oil. As a control, a hydrogel was also prepared with 10% pea protein, 0.48% κ-carrageenan, and 0.08% KCl. All gels were kept at 4 °C for 24 h before analysis.

### 4.3. SEM

SEM micrographs were obtained using Mira3 XMU scanning electron microscope (Tescan Inc., Brno, Czech Republic). Before imaging, the samples underwent lyophilization (for 18 h at 1 mbar, with condenser temperature set to −55 °C) with an Alpha 1-4 LSC system (Martin Christ, Osterode am Harz, Germany). The oil was removed using petroleum ether, followed by vacuum drying at 50 °C, as described by Li et al. [63]. The dried specimens were mounted on aluminum stubs and coated with a 5 nm gold layer using a Quorum Q150 Au sputter coater (Quorum Technologies, Laughton, UK). Imaging was performed at a 10 mm working distance under an accelerating voltage of 10 kV, using a back-scattered electron (BSE) detector to capture representative micrographs at 500x magnification.

### 4.4. Particle Size Distribution (PSD)

PSD was measured using a Mastersizer 2000 laser diffraction analyser (Malvern, Worcestershire, UK). The refractive indices were set to 1.47 for oil and 1.33 for water. EFG samples were added directly into the measurement cell under continuous stirring until an obscuration rate of 10% was reached.

To determine the flocculation index (FI), samples were mixed with a 0.05% SDS solution (1:1 *w*/*w*) to disperse the flocculated particles before measurement. Resulting dispersion was then added to the measurement cell until an obscuration of 10% was achieved. The FI was calculated as the ratio between D (4,3) measured in water and D (4,3) measured in SDS, following the method described by Grasberger et al. [33].

### 4.5. Interfacial Protein Load

The interfacial protein load was quantified according to a modified method described by Grasberger et al. [64]. Briefly, EFGs were placed into centrifugal tubes (Eppendorf, Hamburg, Germany) and spun at 15,000× *g* for 90 min (D3024, DLAB Scientific Co., Ltd., Beijing, China). After centrifugation, a syringe was used to carefully remove the top cream layer to combine it with SDS solution (0.05%) in a 1:10 (*w*/*w*) ratio. Mixtures were stirred for one hour and then centrifuged again at 15,000× *g* for 90 min to separate the cream and aqueous phases. The aqueous layer was collected, and the protein content was measured using the Bradford assay, with bovine serum albumin (BSA) as a standard. The protein surface load was calculated using the following formula:

(1) $$Protein\;surface\;load\;=\frac{C\times V_{tot}}{V_{oil}}\times \frac{D[3,2]}{\phi },\;(mg/m^{2})$$

where *C* represents the concentration of adsorbed proteins (mg/mL); *V_tot_* is the total volume of cream and SDS mixture (mL), *V_oil_* denotes the oil volume after evaporation of water, which was achieved by drying the cream phase under 120 °C. The surface-weighted mean diameter (D(3,2)) was obtained from particle size distribution measurement in SDS, and *ϕ* indicates the oil phase volume fraction.

### 4.6. Rheology

The rheological behavior of gels was analysed using an MCR 92 rheometer (Anton Paar, Graz, Austria) equipped with a parallel plate geometry (PP25, 1 mm gap). All tests were performed at 25 °C within the linear viscoelastic region (LVR).

#### 4.6.1. Frequency and Temperature Sweeps

The frequency sweep was examined at a constant strain of 0.1% over a frequency range from 1 to 100 rad/s. For temperature sweeps, freshly prepared samples were tested to monitor changes in the storage modulus (G′) and loss modulus (G″) at 1% strain as the temperature decreased from 95 °C to 25 °C at a rate of 5 °C/min. The samples were then held at 25 °C for 10 min. To minimize evaporation during testing, silicone oil and a solvent trap were applied.

#### 4.6.2. Strain-Dependent Viscoelasticity

The extent of the linear viscoelastic region (LVR) and changes in viscoelasticity were determined by measuring G′ and G″ over a strain range of 0.01% to 1000% at an oscillation frequency of 1 Hz. The critical strain was defined as the point where G′ decreased by more than 5% from its prior value [43,65].

#### 4.6.3. Shear Yield Stress

Shear yield stress was assessed following to the method described by Yu et al. [66]. Samples were subjected to a flow sweep ranging from 0.001 to 0.1 s^−1^, and the yield stress was defined as the maximum shear stress (peak value) observed during the test.

### 4.7. Texture Analysis

The hardness, chewiness, adhesiveness, cohesiveness, and springiness of the samples were measured using a TA.XTplus Texture Analyzer (Stable Micro Systems, Godalming, UK) equipped with a P20 measuring system. Two compression cycles were carried out at a constant speed of 1 mm/s and a compression ratio of 50%. Tests were performed on cylindrical gel samples (26 mm diameter and 20 mm height).

### 4.8. IDDSI

To assess the suitability of the gels for the dysphagia diet, a fork pressure test was conducted following International Dysphagia Diet Standardization (IDDSI) guidelines. In this test, a fork was pressed onto the gel using the thumb until the thumbnail turned pale, indicating a pressure equivalent to tongue pressure during swallowing (~17 kPa). If the sample maintained its shape after removal of the fork, it was classified as level 6.

### 4.9. In Vitro Digestion

The in vitro digestibility of the samples was assessed following the INFOGEST standardized gastrointestinal digestion protocol [67]. Enzymes used included pepsin for the gastric phase and pancreatin with lipase for the intestinal phase. Enzyme solutions were prepared on the day of the experiment and were kept on ice until use. Digestions were conducted in a shaking water bath at 37 °C and 80 rpm (GFL 1092, GFL, Burgwedel, Germany).

For the oral phase, the gel samples were first minced by manually passing them through a stainless-steel mesh (mesh size 2 mm) to obtain uniformly sized fragments. Subsequently, 5 g of the minced sample was mixed with 5 mL of simulated salivary fluid (CaCl_2_·2H_2_O, 1.5 mM; KCl, 15.1 mM; MgCl_2_·6H_2_O, 0.15 mM; (NH_4_)_2_CO_3_, 0.06 mM; KH_2_PO_4_, 3.7 mM). Five glass beads were added, followed by a 3 min incubation. Next, 10 mL of simulated gastric fluid (CaCl_2_·2H_2_O, 0.15 mM; KCl, 6.9 mM; (NH_4_)_2_CO_3_, 0.5 mM; KH_2_PO_4_, 0.9 mM; MgCl_2_·6H_2_O, 0.12 mM; NaCl, 47.2 mM; pepsin, 2000 U/mL) was introduced to the oral phase digesta, and the mixtures were incubated for 2 h. The pH for the gastric phase was set between 2 and 3, and the final volume was 20 mL. For the intestinal phase, 20 mL of simulated intestinal fluid (KCl, 6.8 mM; CaCl_2_·2H_2_O, 0.6 mM; KH_2_PO_4_, 0.8 mM; MgCl_2_·6H_2_O, 0.33 mM; NaCl, 38.4 mM; pancreatin, 100 U/mL trypsin activity; lipase, 2000 U/mL; bile salts, 10 mM) was mixed with the gastric phase digesta. The pH was adjusted to 7, and the total volume was 40 mL.

For protein digestibility analysis, samples were collected at 0 and 120 min during the gastric phase and at 60, 120, 180, and 240 min during the intestinal phase. After cooling in an ice bath, pepsin activity was inhibited by adjusting the pH to 7. Samples were centrifuged at 6000 rpm for 10 min (MPW-260R, MPW Med. Instruments, Warsaw, Poland), and filtered through a paper filter. The filtered fractions were stored at −25 °C until analysis.

For fat digestibility, unstructured canola oil was used as a control. One milliliter aliquots were collected at 0, 60, 120, 180, and 240 min during the intestinal digestion phase, frozen at −25 °C, and analysed the following day.

### 4.10. Degree of Protein Hydrolysis

The quantification of TCA-soluble peptides and free amino acids was conducted using the fluorescamine method described by Larsen et al. [68]. Briefly, undigested proteins were precipitated by mixing 75 μL of the sample with an equal volume of 24% TCA, followed by incubation for 30 min at −18 °C and centrifugation at 6000 rpm for 20 min (D3024, DLAB Scientific Co., Ltd., Beijing, China). Next, supernatant (30 μL) was combined with 900 μL of borate buffer (0.1 mol/L, pH 8) and 300 μL of fluorescamine-acetone solution (0.2 mg/mL). For fluorescence detection, 250 μL mixtures were transferred to a black 96-well microplate, and readings were taken using a FLUOstar Omega (BMG LABTECH, Ortenberg, Germany) with excitation and emission wavelengths of 390 nm and 480 nm, respectively. A calibration curve was prepared using leucine standards of known concentrations. The degree of protein hydrolysis (DH) was as follows:

(2) $$\;\;\;\;DH\;=\frac{{AG}_{t\;}\times DF}{{AG}_{total\;}}\times 100,\left(\%\right)\;\;\;$$

where *AG_t_* represents the concentration of free α-amino groups in the digesta at a specific digestion time, *DF* is the dilution factor, and *AG_total_* denotes the total concentration of α-amino groups present in the fully hydrolyzed sample (acid hydrolysis in 6N HCl for 18 h).

### 4.11. Free Fatty Acids Determination

To eliminate protein interference, 10 µL of Carrez I and Carrez II reagents were added to 1 mL of digesta collected in Eppendorf tubes. The samples were vortexed, left to stand for 10 min to allow protein precipitation, and then centrifuged at 6000 rpm for 10 min (D3024, DLAB Scientific Co., Ltd., Beijing, China).

Free fatty acid (FFA) release in the digesta was quantified using a fluorimetric Free Fatty Acids Assay Kit (MAK466, Sigma-Aldrich, Steinheim, Germany), following the manufacturer’s instructions. The filtered samples were diluted tenfold with assay buffer, and 10 µL of the diluted mixture was transferred into a black 96-well plate. Then, 90 µL of working reagent was added to each well, and the plate was incubated at room temperature for 30 min. Fluorescence was recorded at λ_Ex_ = 530 nm/λ_Em_ = 585 nm using a FLUOstar Omega microplate reader (BMG LABTECH, Ortenberg, Germany). FFA concentrations were determined using a calibration curve generated from palmitic acid standard solutions of known concentrations.

### 4.12. CLSM

Structural changes during digestion were visualised using a confocal laser scanning microscope (OlympusFLUOVIEW FV1000, Olympus, Tokyo, Japan) equipped with a 10× objective lens. The oil phase was pre-stained with Nile Red by dissolving the dye at a concentration of 1 mg/mL in the heated oil phase during sample preparation.

For digested samples, 10 µL of digesta was combined with 10 µL of FITC dye solution (1 mg/mL) to label the proteins. The mixture was incubated in the dark for 30 min and then 10 µL of the stained sample was placed on a microscope slide and covered with a coverslip. For undigested gels, a small, thin slice of the sample was positioned on a slide, and 10 µL of FITC dye (1 mg/mL) was added. After 30 min of incubation in the dark, a coverslip was carefully pressed onto the sample.

Fluorescence excitation wavelengths were set to 488 nm for the FITC and 543 nm for Nile Red. Micrographs were analysed using ImageJ software (version 1.54g, National Institutes of Health, Bethesda, MD, USA).

### 4.13. Statistical Analysis

All experiments were conducted in triplicate, and results are expressed as mean values ± standard deviation. Statistical differences among samples were assessed using *t*-test, with significance established at *p* < 0.05. Data processing and visualization were performed using Graph Pad Prism software (version 8.0, GraphPad Software, San Diego, CA, USA).

## Figures and Tables

**Table 1:** Texture profile analysis and IDDSI classification of EFGs and HG.

	Hardness, g	Chewiness, g	Adhesiveness, g·s	Springiness	Cohesiveness	IDDSI
EFG-PP	509.43 ± 8.91 ^a^ *	105.28 ± 0.97 ^a^	−72.77 ± 0.95 ^a^	0.69 ± 0.02 ^a^	0.30 ± 0.01 ^a^	Level 6
EFG-PP/LEC	428.94 ± 12.45 ^b^	85.79 ± 0.21 ^b^	−79.59 ± 1.04 ^b^	0.80 ± 0.02 ^b^	0.25 ± 0.01 ^b^	Level 6
EFG-PP/MDG	494.17 ± 12.18 ^ac^	89.27 ± 1.04 ^c^	−91.43 ± 1.02 ^c^	0.72 ± 0.01 ^a^	0.25 ± 0.02 ^b^	Level 6
HG	489.44 ± 9.75 ^c^	48.99 ± 0.77 ^d^	−41.78 ± 1.14 ^d^	0.67 ± 0.02 ^a^	0.15 ± 0.01 ^d^	Level 6

* Different letters indicate significant differences (*p* < 0.05).

## Data Availability

The original contributions presented in this study are included in the article. Further inquiries can be directed to the corresponding author.

## Supplementary Materials

The following supporting information can be downloaded at: https://www.mdpi.com/article/10.3390/gels12040342/s1, Table S1: The IDDSI tests of EFGs and HG.

## Abbreviations

The following abbreviations are used in this manuscript:

BSA	Bovine serum albumin
CLSM	Confocal laser scanning microscopy
DH	Degree of hydrolysis
EFGs	Emulsion-filled gels
FI	Flocculation index
FITC	Fluorescein-5-isothiocyanate
FFA	Free fatty acids
G′	Storage modulus
G″	Loss modulus
HG	Hydrogel
IDDSI	International Dysphagia Diet Standardisation Initiative
κ-CAR	Kappa-carrageenan
LEC	Lecithin
LVR	Linear viscoelastic region
MDG	Mono- and diglycerides of fatty acids
PP	Pea proteins
PSD	Particle size distribution
SDS	Sodium dodecyl sulphate
SEM	Scanning electron microscopy
TCA	Trichloroacetic acid
TPA	Texture profile analysis
