# Supplementary material for: In Vitro Digestibility and Structural Evaluation of Pea Protein-Based Emulsion-Filled Gels Designed for Dysphagia-Friendly Nutrition
Source: Gels. 2026 Apr 19;12(4):342. doi: 10.3390/gels12040342 (PMC13115537; doi:10.3390/gels12040342)
Supplement: Supplementary file 1 [file gels-12-00342-s001.zip › gels-4247241-supplementary.pdf]

Table S1. The IDDSI tests of EFGs and HG.

	Fork pressure test			IDDSI category
**HG**	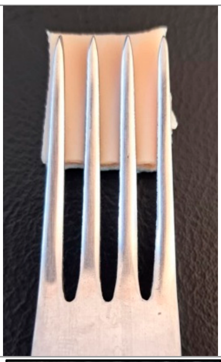	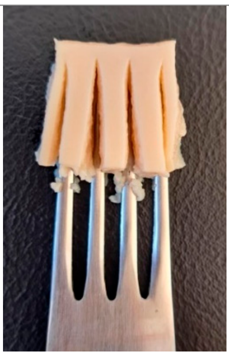	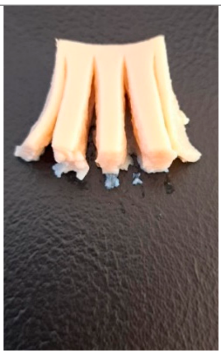	Level 6
**EFG-PP**	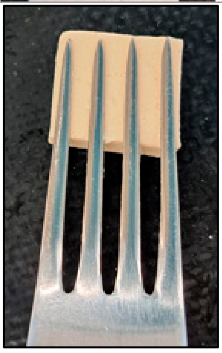	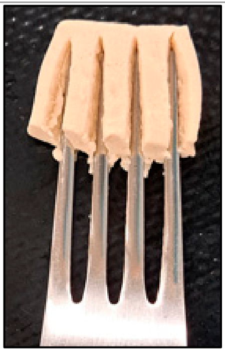	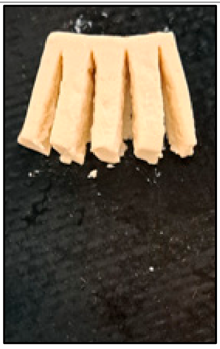	Level 6
**EFG-PP/LEC**	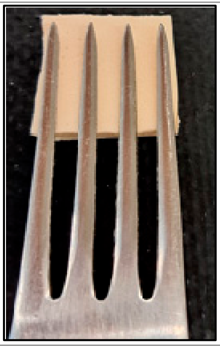	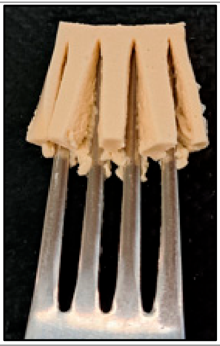	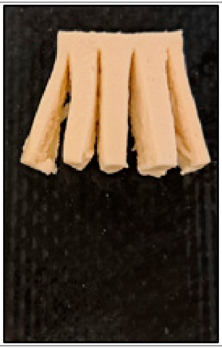	Level 6
**EFG-PP/MDG**	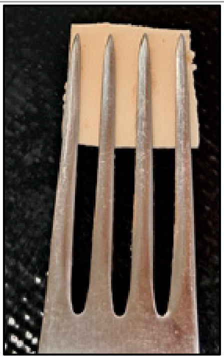	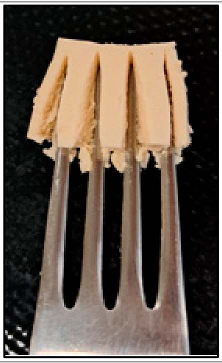	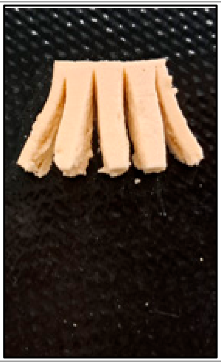	Level 6
